# Is long-term exposure to air pollution associated with poor sleep quality in rural China?

**DOI:** 10.1016/j.envint.2019.105205

**Published:** 2019-12

**Authors:** Gongbo Chen, Hao Xiang, Zhenxing Mao, Wenqian Huo, Yuming Guo, Chongjian Wang, Shanshan Li

**Affiliations:** a Department of Global Health, School of Health Sciences, Wuhan University, Wuhan, Hubei, China; b Department of Epidemiology and Biostatistics, College of Public Health, Zhengzhou University, Zhengzhou, Henan, China; c Department of Epidemiology and Preventive Medicine, School of Public Health and Preventive Medicine, Monash University, Melbourne, Australia

## Abstract

**Background:**

Poor sleep quality is associated with poor quality of life and may even lead to mental illnesses. Several studies have indicated the association between exposure to air pollution and sleep quality. However, the evidence is very limited in China, especially in rural areas.

**Methods:**

Participants in this study were obtained from the Henan Rural Cohort established during 2015–2017. Sleep quality was evaluated using the Pittsburgh Sleep Quality Index (PSQI) in the baseline survey. Poor sleep quality was defined by the global score of PSQI > 5. Participants’ exposures to PM_2.5_, PM_10_ (particulate matter with aerodynamic diameters ≤2.5 μm and 10 μm, respectively) and NO_2_ (nitrogen dioxide) during the three years before the baseline survey were estimated using a satellite-based prediction. The associations between long-term exposure to air pollutants and sleep quality were examined using both the linear regression and logistic regression models.

**Results:**

The IQRs (interquartile range) of mean levels of participants’ exposures to PM_2.5_, PM_10_ and NO_2_ were 3.3 µg/m^3^, 8.8 µg/m^3^, and 4.8 µg/m^3^, respectively. After adjusted for potential confounders, the global score of PSQI (and 95%CI, 95% confidence intervals) increased by 0.16 (0.04, 0.27), 0.09 (−0.01, 0.19) and 0.14 (0.03, 0.24), associated with per IQR increase in PM_2.5_, PM_10_ and NO_2_, respectively. The odds ratios (and 95%CI) of poor sleep quality associated with per IQR increase in PM_2.5_, PM_10_ and NO_2_ were 1.15 (1.03, 1.29), 1.11 (1.02, 1.21) and 1.14 (1.03, 1.25), respectively.

**Conclusions:**

Long-term exposures to PM_2.5_, PM_10_ and NO_2_ were associated with poor sleep quality in rural China. Improvement of air quality may help to improve sleep quality among rural population of China.

## Highlights

• Long-term exposure to PM_2.5_ was associated with poor sleep quality (OR = 1.15).
• Long-term exposure to NO_2_ was associated with poor sleep quality (OR = 1.14).
• Exposure to air pollution showed adverse effects on sleep quality in rural China.
• Health effects of air pollution in rural areas should be given more attention.

## 1 Introduction

Sleep is important for human to maintain health and well-beings. Poor sleep quality is associated with adverse health outcomes, such as cardiovascular disease, diabetes and obesity (Cappuccio et al., 2008, Cappuccio et al., 2011, Okubo et al., 2014). As poor sleep quality can impact memory, response time and ability to concentrate, it also leads to poor quality of life and even mental illnesses (Maglione et al., 2014, Stranges et al., 2012). Previous studies have reported that some demographic and environmental factors and lifestyle habits were associated with poor sleep quality, including socioeconomic status, noise, drinking and smoking (Grandner et al., 2010, Krueger and Friedman, 2009, Miedema and Vos, 2007). Apart from those factors, some studies have indicated that exposure to air pollution had adverse effect on sleep quality (Fang et al., 2015, Zanobetti et al., 2010). For instance, a multicenter cohort study conducted in the U.S. reported exposure to PM_10_ (particulate matter with aerodynamic diameters ≤10 μm) was significantly associated with decreased sleep efficiency (Zanobetti et al., 2010). A cross-sectional study conducted in northeastern China stated long-term exposures to ambient particulate matter (PM) and gaseous pollutants increased the risk of sleep disorder among children (Lawrence et al., 2018).

China is bearing the highest burden of disease due to air pollution in the world (Cohen et al., 2017). Despite numerous studies on health effects of air pollution in China, evidence is still limited for air pollution and sleep quality and inconsistent results of existing studies remain. Moreover, most studies of air pollution and health were conducted in urban areas, but much fewer in rural areas. Although a declined trend has been witnessed during the past decades, China still has a large size of rural population (Liu et al., 2017). Due to socioeconomic factors, health behaviors and availability of health service, rural population may be more vulnerable to environmental exposures (Hartley, 2004).

Based on the established cohort study in rural areas of Henan Province, China, sleep quality of participants was evaluated and their historical exposure to air pollution was estimated using a satellite-based prediction. We aim to examine the relationship between long-term air pollution and poor sleep quality in this study.

## 2 Methods

### 2.1 Study design

**Fig. 1:**
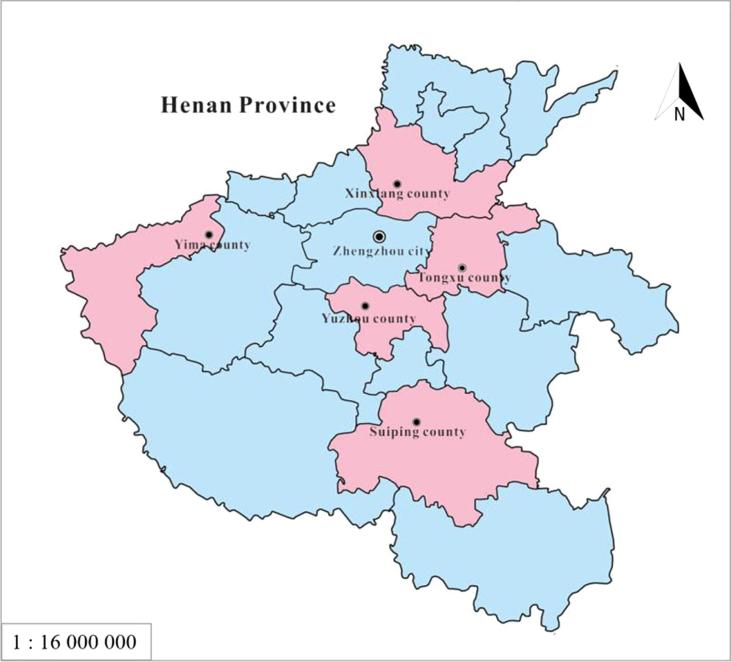
Locations of 5 sampling sites of the Henan Rural Cohort.

The Henan Rural Cohort, established during 2015–2017, is a prospective study of chronic non-communicable disease in Henan Province, China. It has been registered at the Chinese Clinical Trial Registry (Registration number: ChiCTR-OOC-15006699, http://www.chictr.org.cn/showproj.aspx?proj=11375). The profile of this cohort has been previously reported (Liu et al., 2019). In brief, participants of the cohort were selected from rural residents living in 5 counties of Henan Province using a multi-stage stratified cluster sampling strategy, including Xinxiang, Yima, Tongxu, Yuzhou and Suiping counties. The locations of these counties are shown in Fig. 1. In total, 39,259 rural residents aged 18–79 years were recruited in the cohort. Baseline survey was conducted from July 2015 through to September 2017. Data were collected by well-trained medical workers, including demographic and socioeconomic information, health behaviors, disease history, mental health status, and sleep quality.

This study was approved by “Zhengzhou University Life Science Ethics Committee” [Ethics approval code: [2015] MEC (S128)]. Written informed consent was obtained from all participants.

### 2.2 Sleep quality assessment

**Fig. 2:**
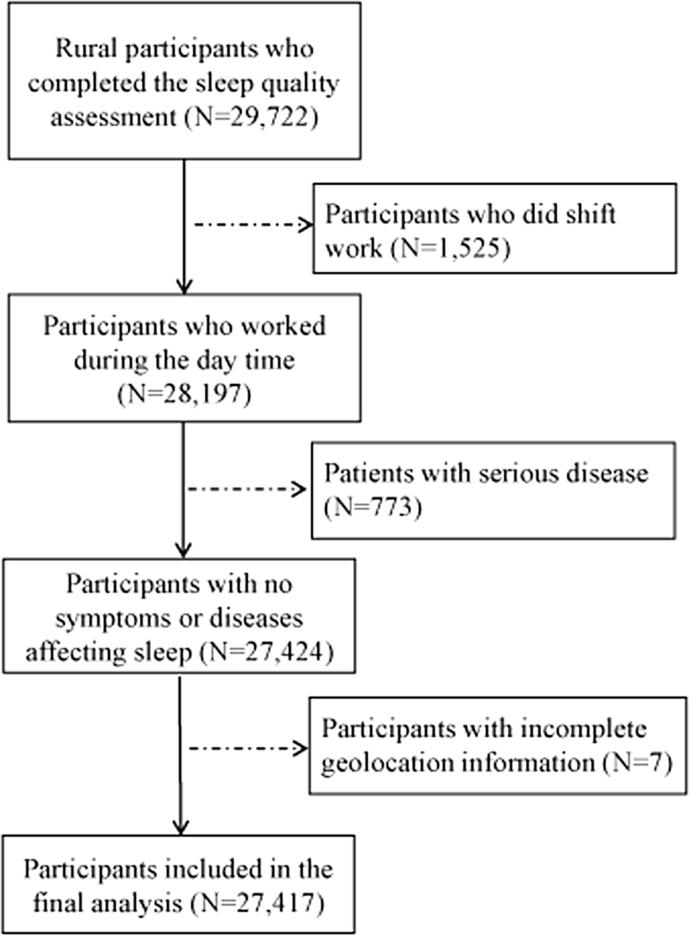
Flowchart of participant selection.

Details of sleep quality assessment have been reported in our previous works (Liu et al., 2018, Wang et al., 2019, Zhang et al., 2019). Briefly, sleep quality for participants from 4 counties (except for Yuzhou county) was evaluated using the Pittsburgh Sleep Quality Index (PSQI). PSQI is a frequently used and suitable instrument to assess sleep quality. It consists of 7 domains and 19 items with the global score between 0 and 21. The higher PSQI score indicates poorer sleep quality and vice versa. The Chinese version of PSQI has been used by previous studies showing high reliability (Tang et al., 2017, Tsai et al., 2005). In addition, we classified all participants into two groups according to the commonly used cut-off global score of 5 (Hinz et al., 2017). Global score > 5 was regarded as poor sleep quality and global score ≤ 5 as good sleep quality. Previous study reported that the cut-off had a sensitivity of 89.6% and specificity of 86.5% (Buysse et al., 1989). In the baseline survey, 29,722 participants from 4 counties completed the sleep quality assessment. Participants who did shift work (n = 1525), had serious disease (e.g., cancer, kidney and heart failure and chronic obstructive pulmonary disease) (n = 773), or had incomplete geolocation information (n = 7) were excluded. As a result, 27,417 participants were included in the final analyses (Fig. 2).

### 2.3 Exposure assessment

In our previous work, concentrations of PM_2.5_ (particulate matter with aerodynamic diameters ≤2.5), PM_10_ and NO_2_ (nitrogen dioxide) were estimated across China with a spatial resolution of 0.1 degree (≈10 km) (Chen et al., 2018a, Chen et al., 2018c, Zhan et al., 2018). A random forests model (machine learning algorithms) and various spatial and temporal predictors were used for estimation, including ground measurements of air pollutants, satellite remote sensing, meteorological data and land use information. Details of data sources, data processing and modeling are shown in the Supplementary Material. Participants’ exposures to three air pollutions during the three years prior to the baseline survey were estimated according to their geolocation information (longitude and latitude of the home address) and date of survey. Daily estimation of concentration of air pollution was aggregated into three-year average.

### 2.4 Statistical analysis

We employed multivariable linear regression model to examine the association between the global score of PSQI and three-year exposures to air pollutants. A range of potential confounders were adjusted, including age (<40, 40–60 or ≥ 60 years), gender (male or female), BMI (Body Mass Index, <24, 24–28 or ≥28 kg/m^2^), educational attainment (primary school or illiteracy, junior high school, or high school or above), smoking (never smoking, quit smoking or current smoking), drinking (never drinking, quit drinking or current drinking), physical activity intensity (low, moderate or high) and income (<500, 500–100 or ≥1000 RMB per month). In addition, we included an indicator of county to control for the potential regional difference. We firstly developed a crude model by only including one air pollutant and an indicator of county, and then developed an adjusted model by including all other covariates as listed above. Apart from the linear regression model, we also used a logistic regression model to examine the association between exposure to air pollution and the prevalence of poor sleep quality (PSQI > 5). The same co-variables were included in the logistic regression model as in the linear regression model, and similarly both crude and adjusted models were developed.

Two-pollutant models were developed by including NO_2_ and one particulate matter pollutant (PM_2.5_ or PM_10_). The potential modification effects of gender, age and BMI were examined by adding an interaction term into the adjusted model. The associations between each single year exposure and sleep quality were also examined during the past three years. Considering meteorological conditions may have impacts on mental health (Berry et al., 2010, Vida et al., 2012), sensitivity analyses were performed by controlling ambient temperature and relative humidity in the model using natural cubic splines (3 degrees of freedom).

All results were expressed as increased global score of PSQI [and 95% confidence intervals (95%CIs)] or odds ratio of poor sleep quality associated with per IQR (interquartile range) increase in level of each pollutant. All statistical analyses were performed using R software (version 3.3.3, R Development Core Team 2015).

## 3 Results

A summary of participants’ demographic characteristics and health behaviors is shown in Table 1. Of all participants, 5911 (21.6%) showed poor sleep quality according to the cut-off global score of 5. Higher mean age (55.8 *vs.* 55.2 years) and higher fractions of female (72% *vs.* 57%), low educational attainment participants (55% *vs.* 43%) were observed among those with poor sleep quality than others. In addition, participants with poor sleep quality tended to be non-smoker, non-drinkers and had lower income. Other factors were distributed evenly among these two groups. A summary of participants’ global score of PSQI and levels of their historical exposures to three pollutants is show in Table 2. The mean global score was 3.8, ranging from 0 to 20. The mean levels of exposures to PM_2.5_, PM_10_ and NO_2_ were 72.3 µg/m^3^, 130.0 µg/m^3^ and 38.2 µg/m^3^, respectively, with the IQRs of 3.3 µg/m^3^, 8.8 µg/m^3^, and 4.8 µg/m^3^, respectively.

**Table 1:** Demographic characteristics and health behaviors of all participants.

Factors	Participants with poor sleep quality		Participants with good sleep quality	
	n	%	n	%
Age (years, Mean ± SD)	58.4 ± 10.7	–	55.2 ± 12.5	–
Gender				
Male	1670	28%	9239	43%
Female	4241	72%	12,261	57%
BMI (kg/m^2^)				
<24	2603	44%	9431	44%
24–28	2289	39%	8387	39%
>28	1001	17%	3621	17%
Education attainment				
Primary school or illiteracy	3258	55%	9235	43%
Junior high school	2001	34%	8520	40%
High school or above	652	11%	3745	17%
Smoking				
Never smoking	4719	80%	15,243	71%
Quit smoking	345	6%	1767	8%
Current smoking	847	14%	4490	21%
Drinking				
Never drinking	4949	84%	16,527	77%
Quit drinking	246	4%	1081	5%
Current drinking	716	12%	3892	18%
Physical activity				
Low	1804	31%	6790	32%
Moderate	2331	39%	7797	36%
High	1776	30%	6913	32%
Income (RMB per month)				
<500	2454	42%	7657	36%
500–1000	1801	30%	6871	32%
>1000	1656	28%	6972	32%
Total	5911	100%	21,500	100%

SD: standard deviation; BMI: body mass index.

**Table 2:** Summary of participants’ global score of PSQI and levels of exposures to three air pollutants (µg/m^3^) during the three years prior to the survey.

PSQI or Pollutants	Mean	Min	Quantiles			Max	IQR
			Q25	Q50	Q75		
Global score of PSQI	3.8	0.0	2.0	3.8	5.0	20.0	3.0
PM_2.5_	72.3	68.0	70.5	71.8	73.8	79.3	3.3
PM_10_	130.0	122.4	125.5	128.6	134.3	143.5	8.8
NO_2_	38.2	31.0	35.9	37.5	40.7	47.4	4.8

PSQI: Pittsburgh Sleep Quality Index; IQR: interquartile range; PM_2.5_: particulate matter with aerodynamic diameters ≤2.5 μm; PM_10_: particulate matter with aerodynamic diameters ≤10 μm; NO_2_: nitrogen dioxide.

**Fig. 3:**
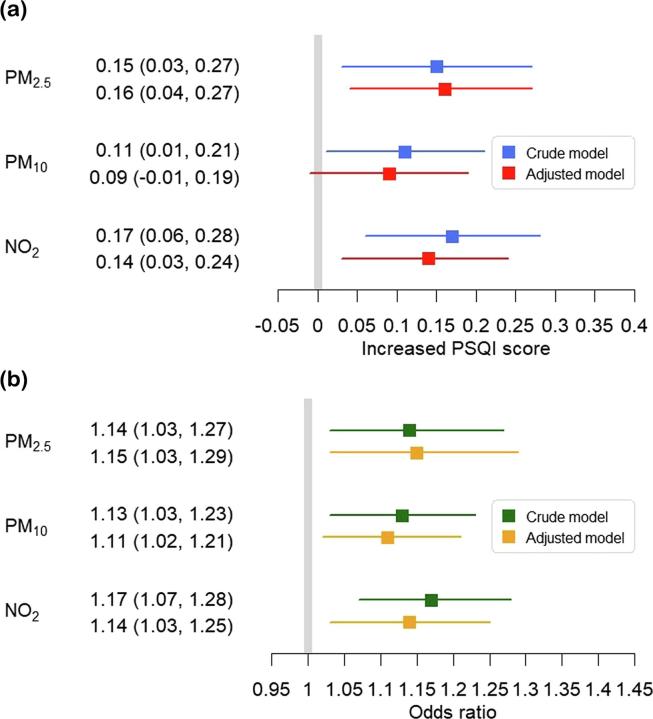
Increased PSQI and odds ratio of poor sleep quality (and 95%CI) associated with per IQR increase in level of exposure to air pollution.

The associations between long-term exposures to air pollutants and sleep quality are shown in Fig. 3. The results of crude linear regression models showed higher global score of PSQI associated with increased level of air pollution. After adjusted for potential confounders, the global scores of PSQI (and 95%CIs) increased by 0.16 (0.04, 0.27), 0.09 (−0.01, 0.19) and 0.14 (0.03, 0.24) associated with per IQR increase in PM_2.5_, PM_10_ and NO_2_, respectively.

The results of adjusted logistic regression models showed that the odds ratios of poor sleep quality (and 95%CIs) associated with per IQR increase in PM_2.5_, PM_10_ and NO_2_ were 1.15 (1.03, 1.29), 1.11 (1.02, 1.21) and 1.14 (1.03, 1.25), respectively. The results of crude models were consistent with those of adjusted models. The concentration-response curves for the association between long-term air pollution and sleep quality are shown in Fig. S1 in the Supplementary Material.

The results of two-pollutant models for the association between long-term air pollution and sleep quality are shown in Table 3. After adjusted for the effect of PM_2.5_ or PM_10_, both linear and logistic regression models revealed higher effect estimates of NO_2_. The global scores of PSQI (and 95%CIs) increased by 0.50 (0.23, 0.78) and 0.54 (0.19, 0.89) associated with per IQR increase in NO_2_ after controlling for PM_2.5_ or PM_10_, respectively, and the corresponding odds ratios (and 95%CIs) of poor quality were 1.45 (1.12, 1.88) and 1.89 (1.02, 3.48), respectively. Weak and insignificant associations between PM_2.5_ or PM_10_ and sleep quality were observed.

**Table 3:** Results of multi-pollutant models for the association between long-term air pollution and sleep quality.

Models	Increased PSQI or OR (95%CI)		
	PM_2.5_	PM_10_	NO_2_
Linear regression models			
PM_2.5_ + NO_2_	−0.11 (−0.30, 0.08)	…	0.50 (0.23, 0.78)
PM_10_ + NO_2_	…	−0.16 (−0.47, 0.15)	0.54 (0.19, 0.89)
Logistic regression models			
PM_2.5_ + NO_2_	0.94 (0.78, 1.13)	…	1.45 (1.12, 1.88)
PM_10_ + NO_2_	…	0.95 (0.71, 1.29)	1. 89 (1.02, 3.48)

Note: Increased PSQI and OR of poor sleep quality were associated with per interquartile range increase in concentration of each pollutant.

PSQI: Pittsburgh Sleep Quality Index; OR: odds ratio; 95%CI: 95% confidence interval; PM_2.5_: particulate matter with aerodynamic diameters ≤2.5 μm; PM_10_: particulate matter with aerodynamic diameters ≤10 μm; NO_2_: nitrogen dioxide.

The results of interaction analyses are shown in Table 4. Stronger associations between long-term air pollution and sleep quality were presented among male participants and those aged <40 years. No remarkable interaction effect was observed for BMI. The results for temporal variation in the association are shown in Table 5. Stronger associations between long-term air pollution and sleep quality were observed during the 1st year than the 2nd and 3rd years prior to the baseline survey. The results of sensitivity analyses showed controlling ambient temperature and relative humidity did not change the results substantially.

**Table 4:** Results of stratified analyses for the association between long-term air pollution and sleep quality.

	Increased PSQI (95%CI)			OR (95%CI)		
Interaction term	PM_2.5_	PM_10_	NO_2_	PM_2.5_	PM_10_	NO_2_
Gender						
Male	0.59 (0.37, 0.81)	0.61 (0.39, 0.84)	0.66 (0.43, 0.88)	1.35 (1.08, 1.68)	1.40 (1.11, 1.76)	1.50 (1.19, 1.89)
Female	0.32 (0.18, 0.45)**	0.29 (0.17, 0.42)**	0.34 (0.21, 0.47)**	1.23 (1.07, 1.41)	1.22 (1.08, 1.38)*	1.28 (1.12, 1.46)**
Age						
<40	0.39 (0.20, 0.59)	0.38 (0.17, 0.58)	0.45 (0.24, 0.66)	1.26 (1.01, 1.59)	1.30 (1.02, 1.65)	1.42 (1.11, 1.81)
40–60	0.08 (−0.05, 0.22)**	0.01 (−0.10, 0.13)**	0.06 (−0.07, 0.19)**	1.08 (0.96, 1.23)	1.03 (0.92, 1.15)	1.06 (0.95, 1.19)*
≥60	0.16 (0.02, 0.29)**	0.11 (−0.01, 0.23)*	0.15 (0.03, 0.28)**	1.21 (1.07, 1.37)	1.16 (1.05, 1.29)	1.18 (1.06, 1.32)
BMI						
<24	0.18 (0.04, 0.31)	0.10 (−0.02, 0.22)	0.11 (−0.02, 0.24)	1.15 (1.01, 1.31)	1.09 (0.98, 1.22)	1.11 (0.98, 1.25)
24–28	0.13 (−0.01, 0.27)	0.07 (−0.05, 0.19)	0.13 (0.01, 0.26)	1.16 (1.02, 1.32)	1.11 (1.00, 1.24)	1.14 (1.02, 1.29)
>28	0.17 (0.01, 0.34)	0.11 (−0.05, 0.27)	0.19 (0.02, 0.36)	1.14 (0.98, 1.34)	1.12 (0.97, 1.30)	1.20 (1.03, 1.40)

Note: *indicates *p* values for interaction <0.05; **indicates *p* values for interaction <0.01. PSQI: Pittsburgh Sleep Quality Index; OR: odds ratio; 95%CI: 95% confidence interval; BMI: body mass index; PM_2.5_: particulate matter with aerodynamic diameters ≤2.5 μm; PM_10_: particulate matter with aerodynamic diameters ≤10 μm; NO_2_: nitrogen dioxide.

**Table 5:** The associations between long-term air pollution and sleep quality during each single year before the baseline survey.

Pollutants	Increased PSQI or OR (95%CI)		
	1st year before the survey	2nd year before the survey	3rd year before the survey
Increased PSQI			
PM_2.5_	0.13 (0.07, 0.19)	0.00 (−0.11, 0.12)	−0.03 (−0.10, 0.03)
PM_10_	0.09 (0.02, 0.16)	−0.01 (−0.12, 0.10)	0.01 (−0.06, 0.07)
NO2	0.06 (−0.06, 0.18)	0.13 (0.01, 0.25)	0.02 (−0.07, 0.11)
OR of poor sleep quality			
PM_2.5_	1.13 (1.07, 1.19)	1.00 (0.89, 1.12)	1.01 (0.95, 1.07)
PM_10_	1.03 (1.03, 1.17)	1.02 (0.92, 1.13)	1.04 (0.98, 1.10)
NO_2_	1.14 (1.02, 1.27)	1.14 (1.02, 1.27)	1.05 (0.97, 1.13)

Note: Increased PSQI and OR of poor sleep quality were associated with per interquartile range increase in concentration of each pollutant. PSQI: Pittsburgh Sleep Quality Index; OR: odds ratio; 95%CI: 95% confidence interval; PM_2.5_: particulate matter with aerodynamic diameters ≤2.5 μm; PM_10_: particulate matter with aerodynamic diameters ≤10 μm; NO_2_: nitrogen dioxide

## 4 Discussion

To the best of our knowledge, this is the first study to examine the effect of long-term air pollution on sleep quality among rural adults. Based on the baseline survey of the Henan Rural Cohort, participants’ sleep quality was evaluated via PSQI and their exposure to air pollution during the three years prior to the survey were estimated. After controlling for potential confounders, it was illustrated that long-term exposures to PM_2.5_, PM_10_ and NO_2_ were associated with increased global score of PSQI and higher risk of poor sleep quality. Stronger effects of NO_2_ and weaker effects of PM on sleep quality were presented in two-pollutant models.

Currently, evidence for adverse effect of air pollution on sleep quality is limited in China or elsewhere in the world. A multi-center cohort study in the U.S. reported an IQR increase (17.4 µg/m^3^) in concentration of PM_10_ was associated with 19.4% increase (95%CI: 3.7%, 37.5%) in percentage of sleep time at less than 90% O_2_ saturation and 1.2% (95%CI: 0.004%, 2.4%) decrease in sleep efficiency during summer time (Zanobetti et al., 2010). Another study conducted in Boston revealed long-term exposure to black carbon was significantly associated with shorter sleep time among male participants and those have low socioeconomic status (Fang et al., 2015). A study conducted in seven northeastern Chinese cities presented long-term exposure to PM_2.5_ increased the risk of sleep disorder among children [OR and 95%CI: 1.47 (1.34, 1.62)] (Lawrence et al., 2018). Similar to previous studies, our study found exposure to air pollution had adverse effect on sleep quality. However, the effect estimates (e.g., OR) are not comparable due to differences in exposure and outcome assessments. Therefore, more studies are needed in the future to provide more solid and robust evidence for the relationship between air pollution and sleep quality.

The biological mechanism for air pollution and sleep quality is far from clear, but previous studies have suggested several potential pathways. PM may have an effect on sleep quality via impacting the central nervous system. Inhaled particles may reach the brain through nose and the olfactory nerve (Elder et al., 2006), which may lead to changed inflammatory responses and neuro-transmitter levels in the brain (Kleinman et al., 2008, Mitsushima et al., 2008). Evidence showed the neural inflammation associated with brain particle deposition may impact sleep-wake cycles (Bertini et al., 2010, Pan et al., 2013). In addition, exposures to PM and NO_2_ have been linked with various respiratory disease, such as asthma and chronic obstructive pulmonary disease (Halonen et al., 2008). Inhaled pollutants may induce inflammation on the upper respiratory system and disrupt the nasal respiratory, and further increase the risk of sleep-disordered breathing (Fang et al., 2015).

In the interaction analyses, it revealed the association between air pollution and sleep quality was modified by age, gender and educational attainment. Stronger effects of air pollution on sleep were observed among males and those aged <40 years. Young male people are main labor force in rural China, who are responsible for most of outdoor works. Considering the remarkable differences in level of air pollution in outdoor and indoor environment (Zhao et al., 2007), young males tend to expose to much higher level of air pollution than females or the elderly (age ≥ 60 years). The strengthened association between air pollution and sleep among male adults was also reported previously (Fang et al., 2015). Apart from air pollution, some risk factors were reported to have impacts on sleep quality (Grandner et al., 2010, Krueger and Friedman, 2009, Miedema and Vos, 2007). Higher prevalence of poor sleep quality among elderly participants (age ≥ 60 years) could be explained by higher exposure to other risk factors, including age, living alone, anxiety and chronic disease (Luo et al., 2013). We infer their sleep quality is less affected by air pollution compared with younger participants.

Participants’ historical exposure to air pollution during the three-year period was estimated using a satellite-based prediction. This method of exposure assessment has been used in our previous studies (Chen et al., 2018b, Li et al., 2019, Yang et al., 2019). We have tried to use the mean levels of pollutants during other exposure periods (e.g., previous one or two years), but the results did not change substantially. The majority of households in rural Henan are one-story houses with kitchen and living areas and yards (Wu et al., 2015), which may have better natural ventilation than urban residential buildings. With rapid industrial and economic development, clean fuels and ventilation facilities are widely used in rural China (Zhang et al., 2013). Moreover, rural residents tend to do more daily outdoor activities and have more exposure to outdoor air pollution than urban residents. Thus, the satellite-based estimation of air pollutants was closely linked to participants’ exposure during the study period.

The baseline survey of the Henan Rural Cohort was conducted in 5 counties. Considering different levels of air pollution in these 5 counties, we included a categorical variable of county in both the crude and adjusted models. We also noted that having sleeping pills also had effect on sleep quality (Veldi et al., 2005), but we did not control this variable in the analysis, as almost no rural resident reported the use of sleeping pills in our study. Exposure to PM_10_ was significantly associated with increased PSQI (linear regression model), but was not with prevalent poor sleep quality (logistic regression). The inconsistency of results was due to converting PSQI into a categorical variable using the cut-off score of 5. Converting continuous variable into categorical variable led to the reduced variability of PSQI. In addition, although a cut-off score of 5 showed high sensitivity and specificity, it is also possible to get some mis-diagnosed cases of poor sleep quality in this study.

Nowadays, due to the rapid urbanization and work force immigration, mental health problem in rural areas has become a big health challenge in China (Gong et al., 2012). However, most of existing studies and policies regarding health effects of air pollution are focused on some better known health outcomes (e.g., cardiovascular and respiratory diseases) based on urban environment. Health issues of rural population related to air pollution, including poor sleep quality and other mental illnesses, have been given little attention. Effective policies should be made to improve mental health focusing on rural population and to mitigate air pollution in rural areas of China.

Several limitations of our study should be noted. The PSQI was used in this study to identify participants with poor sleep quality, however, the exact onset date of their sleep problem was unavailable. We did not analyze the county-specific association, as participants in this study were not evenly located in 4 counties. For example, the results in Yima County was unstable with big uncertainty, due to small sample size. Moreover, exposure assessment in this study represented participants’ exposures to air pollution during the whole day, but poor sleep quality is the behavior at night. The inconsistency in time may lead to exposure misclassification. Future studies should improve the exposure assessment by focusing on night-time exposure.

## 5 Conclusions

This study provides evidence for adverse effect of air pollution on sleep quality in rural China. Poor sleep quality associated with air pollution should be given more attention by policy-makers and the public. However, the association between air pollution and sleep has not been well known. More studies are in need in the future to explore the biological mechanism for the relationship, and to also examine such relationship among different populations and in difference environments.
