# Supplementary material for: Is long-term exposure to air pollution associated with poor sleep quality in rural China?
Source: Environ Int. 2019 Dec;133(Pt B):105205. doi: 10.1016/j.envint.2019.105205 (PMC6853164; doi:10.1016/j.envint.2019.105205)
Supplement: Supplementary data 1 [file mmc1.docx]

**Supplemental Material**

**Is long-term exposure to air pollution associated with poor sleep quality in rural China?**

**Estimating concentrations of PM_2.5,_ PM_10_ and NO_2_**

*Data downloading and processing*

Daily concentrations of PM_2.5_ and PM_10_ across China during 2005-2016 were estimated using MODIS AOD, meteorological data, land use information and other predictors. Daily concentration of NO_2_ was estimated with satellite-derived OMI data (Daily Level-3 Nitrogen Dioxide Product) and other predictors. More details about the data downloading and processing were previously reported (Chen et al. 2018a; Chen et al. 2018b). Daily ground-level measurements of PM_2.5_, PM_10_ and NO_2_ were obtained from 1,479 stations of the China National Environmental Monitoring Center (CNEMC) from May 2014 to December 2016.

*Model development*

We used a machine learning method (random forests) for model development and prediction. This method is user-friendly, as there is no need to define the complex relationships between predictors (e.g., linear or nonlinear relationships and interactions) and the variable importance measures provided by random forests help user to identify important variables and noise variables (Hu et al. 2017). The final model is shown as follows:

*PM_ij_* = *AOD_ij_* + *TEMP_ij_* + *RH_ij_* + *BP_ij_* + *WS_ij_*+ *NDVI_ij_*+ *Urban_cover_ij_* + *doy_i_* + *log(elev_j_)*

*NO_2ij_* = *OMI_ij_* + *TEMP_ij_* + *BP_ij_* + *RH_ij_* + *WS_ij_* + *NDVI_ij_*+ *Urban_cover_ij_* + *doy_i_* + *log(elev_j_)*

where *PM_ij_* is the PM_2.5_ or PM_10_ on day *i* at station *j*; *NO_2ij_* is the NO_2_ on day *i* at station *j*; *AOD_ij_* is the combined AOD; *OMI_ij_* is the satellite-derived OMI value;*TEMP*, *RH*, *BP* and *WS* are mean temperature, relative humidity, barometric pressure and wind speed on day *i*, respectively; *NDVI* is the monthly average NDVI value at station *j*; *Urban_cover* is the percentage of urban cover with a buffer radius of 10 km around station *j*; *doy* is the day of the year; *log(elev_j_)* is the log transformed elevation.

*Model validation and prediction*

To evaluate the predictive ability of the final model, a 10-fold cross-validation (CV) was performed. The results are shown in Table 1.

**Table 1. Results of 10-fold cross-validation for PM_2.5,_ PM_10_ and NO_2_**

| Pollutants | Daily model | |  | Annual averages | |
| --- | --- | --- | --- | --- | --- |
|  | CV R^2^ | RMSE |  | CV R^2^ | RMSE |
| PM_2.5_ | 83% | 18.1 µg/m^3^ |  | 86% | 6.9 µg/m^3^ |
| PM_10_ | 78% | 31.5 µg/m^3^ |  | 81% | 14.4 µg/m^3^ |
| NO_2_ | 64% | 12.4 µg/m^3^ |  | 72% | 6.5 µg/m^3^ |

*RMSE: Root mean square error; CV: cross-validation

The final random forests models were used to predict daily concentration of air pollutants in China. A 0.1-degree (≈ 10 km) grid (including around 96,103 grid cells) covering all of China was created for data integration and prediction. Daily concentrations of PM_2.5,_ PM_10_ and NO_2_ were estimated for each grid cell during the study period. Predicted daily concentrations were aggregated into three-year averages.

**Table S2. Increased PSQI score and odds ratio (OR) of poor sleep quality associated with per IQR increase in each air pollutant controlling for temperature and humidity.**

| Pollutant | Increased PSQI (95%CI) | OR (95%CI) |
| --- | --- | --- |
| PM_2.5_ | 0.16 (0.04, 0.28) | 1.15 (1.02, 1.29) |
| PM_10_ | 0.14 (0.05, 0.24) | 1.13 (1.03, 1.23) |
| NO_2_ | 0.37 (0.19, 0.56) | 1.33 (1.12, 1.57) |

*The mean temperature and relative humidity during the study period were controlled in the model using natural cubic splines (3 degrees of freedom). PSQI: Pittsburgh Sleep Quality Index.


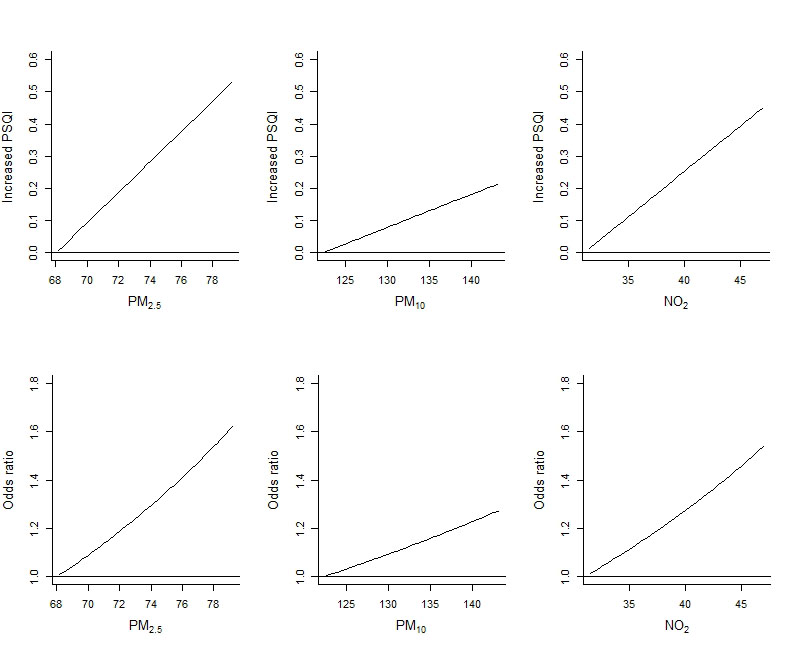


**Figure S1. Concentration-response curve for the association between long-term exposure to air pollutants (µg/m^3^) and sleep quality**

**References:**

Chen G, Li S, Knibbs LD, Hamm N, Cao W, Li T, et al. 2018a. A machine learning method to estimate pm 2.5 concentrations across china with remote sensing, meteorological and land use information. Science of the Total Environment 636:52-60.

Chen G, Wang Y, Li S, Cao W, Ren H, Knibbs LD, et al. 2018b. Spatiotemporal patterns of pm10 concentrations over china during 2005–2016: A satellite-based estimation using the random forests approach. Environmental Pollution 242:605-613.

Hu X, Belle JH, Meng X, Wildani A, Waller LA, Strickland MJ, et al. 2017. Estimating pm2. 5 concentrations in the conterminous united states using the random forest approach. Environmental science & technology 51:6936-6944.
